# Supplementary material for: Asymmetric opening of the homopentameric 5-HT3A serotonin receptor in lipid bilayers
Source: Nat Commun. 2021 Feb 16;12:1074. doi: 10.1038/s41467-021-21016-7 (PMC7887223; doi:10.1038/s41467-021-21016-7)
Supplement: Supplementary file 1 — Supplementary Information [file 41467_2021_21016_MOESM1_ESM.pdf]

Supplementary Information

Asymmetric opening of the homopentameric 5-HT_{3A} serotonin receptor in lipid bilayers

Yingyi Zhang[1,2†‡], Patricia M. Dijkman[1,2†§], Rongfeng Zou[3], Martina Zandl-Lang[4], Ricardo M. Sanchez[1,2], Luise Eckhardt-Strelau[1], Harald Köfeler[5], Horst Vogel[3,6], Shuguang Yuan[3]*, Mikhail Kudryashev[1,2]*

[1]Max Planck Institute of Biophysics, Frankfurt am Main, Germany.
[2]Buchmann Institute for Molecular Life Sciences (BMLS), Goethe University of Frankfurt, Frankfurt am Main, Germany.
[3]Research Center for Computer-Aided Drug Discovery, Shenzhen Institutes of Advanced Technology, Chinese Academy of Sciences, Shenzhen, China.
[4]Department of Pediatrics and Adolescent Medicine, Division of General Pediatrics, Medical University of Graz, Graz, Austria.
[5]Core Facility Mass Spectrometry, ZMF, Medical University of Graz, Graz, Austria.
[6]Institute of Chemical Science and Engineering (ISIC), Ecole Polytechnique Fédérale de Lausanne (EPFL), Lausanne, Switzerland.
[‡]Present address: Biological Cryo-EM Center, Hong Kong University of Science and Technology, Clear Water Bay, Kowloon, Hong Kong, China.
[§]Present address: Max Planck Institute of Biochemistry, Martinsried, Germany; Institute of Neuropathology, University Medical Center Göttingen, Göttingen, Germany; Cluster of Excellence "Multiscale Bioimaging: from Molecular Machines to Networks of Excitable Cells" (MBExC), University of Göttingen, Göttingen, Germany.
[†]These authors contributed equally to this work.
*Correspondence to: M.K. (misha.kudryashev@biophys.mpg.de) or S.Y. (shuguang.yuan@siat.ac.cn).

Contents

Supplementary Fig. 1 – Characterisation of $5HT_3R$-Salipro.
Supplementary Fig. 2 – Data Processing.
Supplementary Fig. 3 – Resolution estimation and model validation.
Supplementary Fig. 4 – Map quality.
Supplementary Fig. 5 – Symmetry expansion and monomer-focussed 3D classification of apo-C1 $5HT_3R$-Salipro data set.
Supplementary Fig. 6 – RMSD and pore radius during MD simulations.
Supplementary Fig. 7 – M2 conformation and pore wetting during MD simulations.

Supplementary Table 1 – Lipid headgroup composition of $5HT_3R$-Salipro.
Supplementary Table 2 – Asymmetry of $5HT_3R$-Salipro models.
Supplementary Table 3 – Comparison between the TMD of the apo-C1, apo-C5, and serotonin-bound $5HT_3R$-Salipro models.
Supplementary Table 4. TMD inter-subunit cavity.
Supplementary Table 5. Details of data collection and model validation.

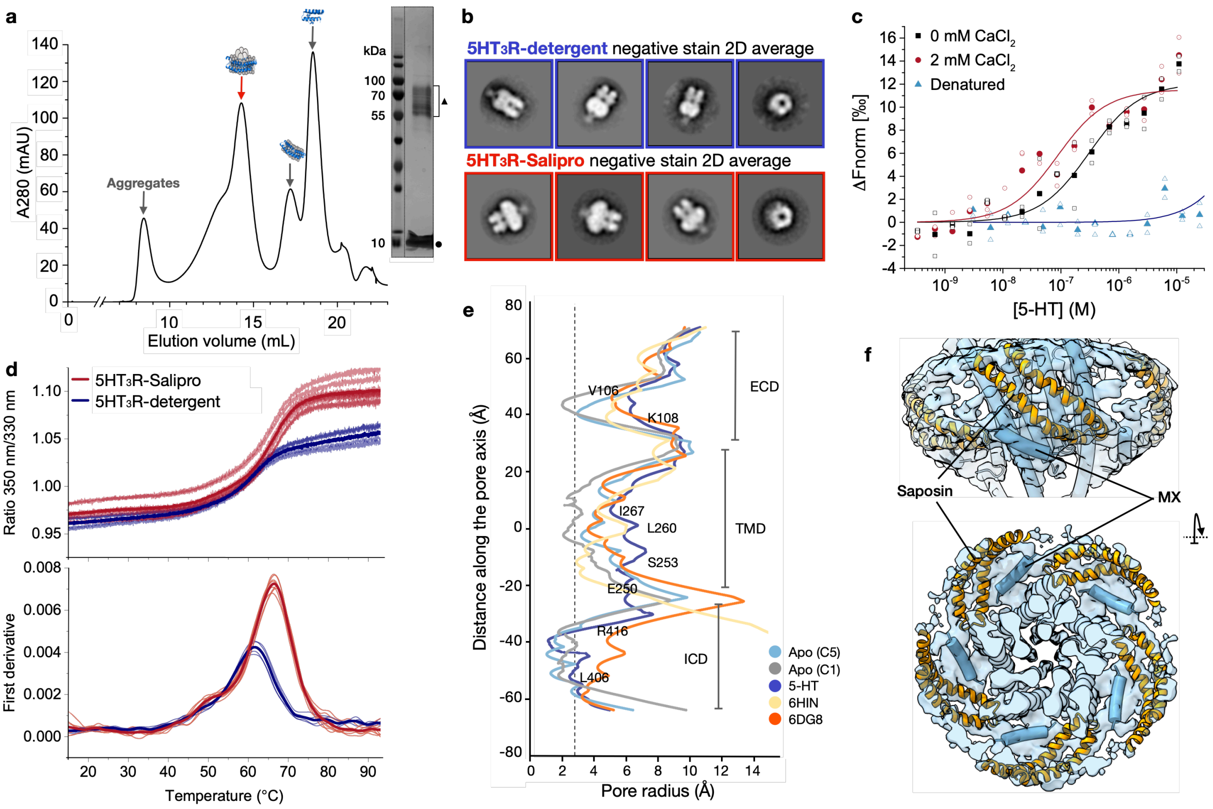

Supplementary Fig. 1. Characterisation of $5HT_3R$-Salipro. a, Gel filtration chromatogram and SDS-PAGE gel for Salipro-reconstituted $5HT_3R$ (saposin, black sphere; $5HT_3R$, black triangle). Comparable results were obtained for at least three further purifications used for lipidomics analysis. **b**, Negative stain 2D class averages of C12E9-solubilised (top) and Salipro-reconstituted (bottom) $5HT_3R$. **c**, Affinity of $5HT_3R$-Salipro for serotonin determined by microscale thermophoresis: dissociation constants of 210±70 nM and 30±10 nM were obtained in absence (black) and presence (red) of 2 mM $CaCl_2$, respectively. Denatured $5HT_3R$-Salipro (blue) as a negative control showed no significant ligand binding. Data shown for two technical replicates; solid markers represent average values, open markers the measured individual data points. **d**, NanoDSF measurements of thermal stability for $5HT_3R$-Salipro (red, apparent melting temperature (T_m) of 66.2±0.2 °C) and detergent-solubilised $5HT_3R$ (dark-blue, apparent T_m=61.5±0.3 °C, both values represent mean ± standard deviation). Thick lines represent average, thin transparent lines individual measurements (two and four biological replicates for detergent-solubilised receptor and $5HT_3R$-Salipro, respectively). **e**, Pore radius plot of the static structures along the receptor pseudo-symmetric axis determined using HOLE[1]. Dashed line represents the radius of a hydrated sodium ion (2.76 Å). **f**, Side and top views of saposin model (orange ribbons, using PDB 6D80 as a model) and apo-C5 model (light blue tubes) fitted into the apo-C5 EM density map (transparent blue surface). The density was partially disordered and not good enough for atomic model building. Source data for panels **a**, **c**, and **d** are provided in the Source Data file.

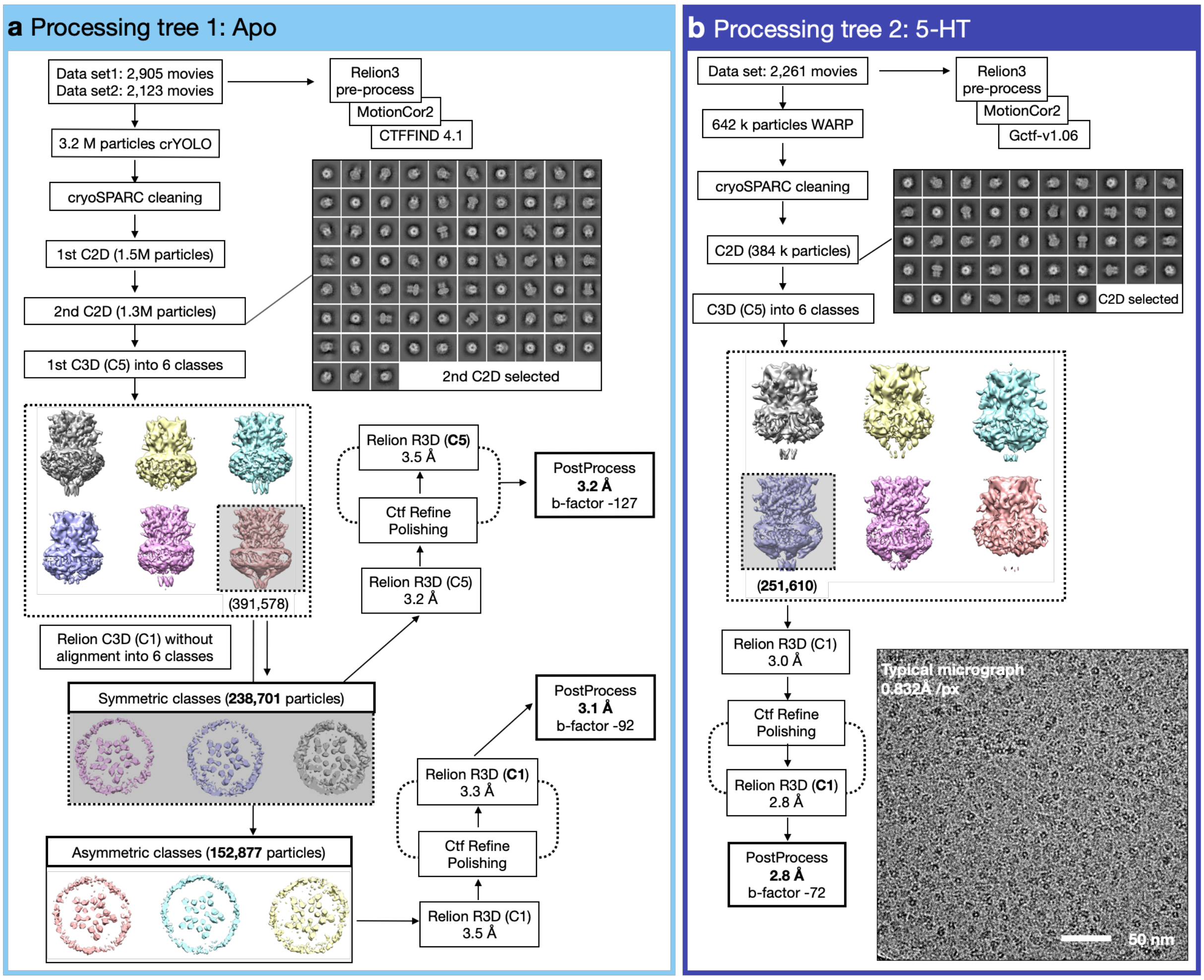

Supplementary Fig. 2. Data Processing. Processing pipeline for the **a**, apo-5HT$_3$R-Salipro, and **b**, serotonin-5HT$_3$R-Salipro data sets.

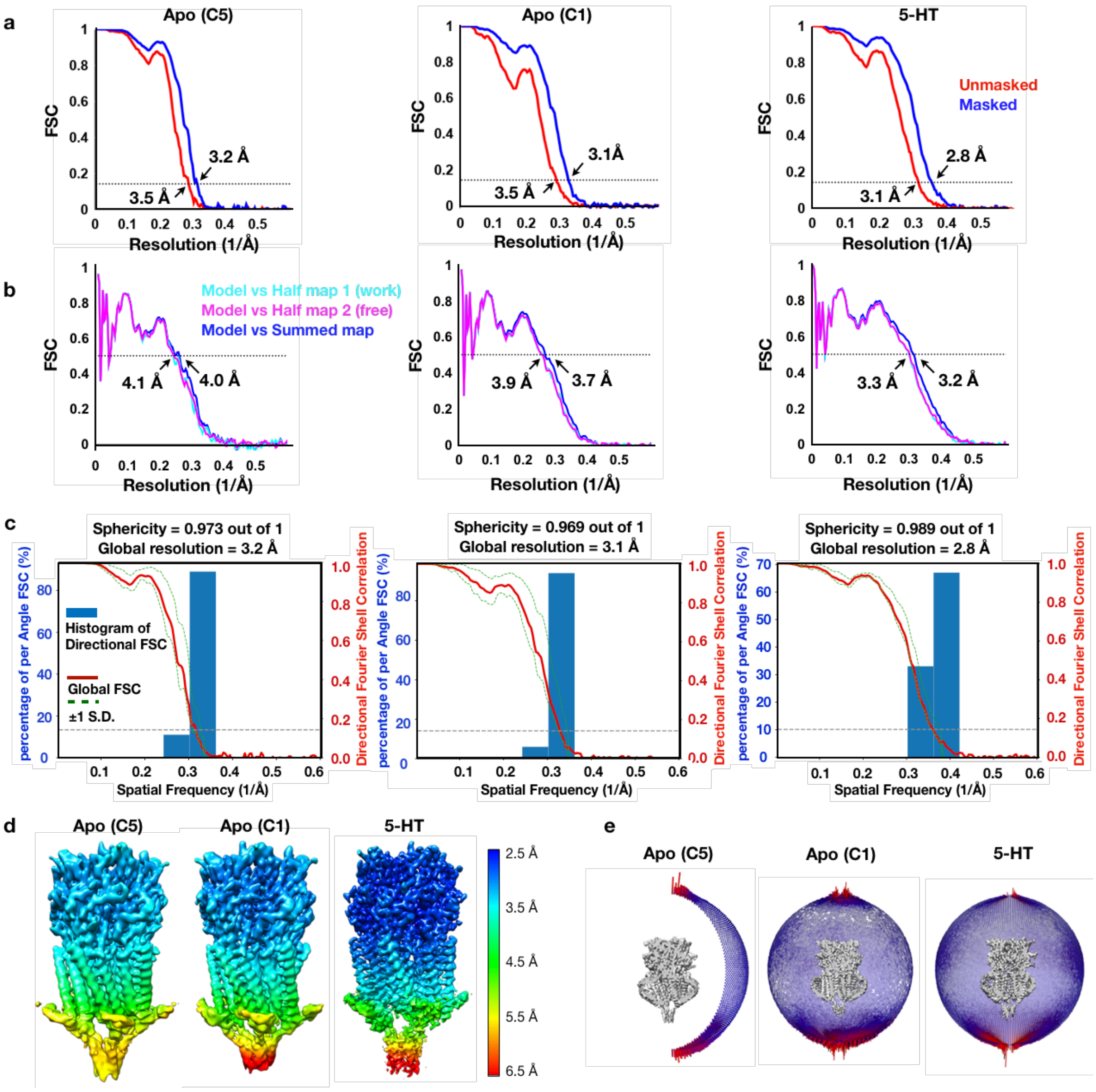

Supplementary Fig. 3. Resolution estimation and model validation. **a**, Fourier shell correlation (FSC) curves for the reconstruction after (blue) and before (red) applying a soft mask in RELION 3.0[2,3]. **b**, FSC curves of the refined built models versus half map 1 (used during refinement, cyan), versus half map 2 (not used during refinement, magenta), and versus summed map (full data set, blue). **c**, 3DFSC[4] data describing the global and directional resolution. **d**, Local map resolution estimated using RELION 3.0[2,3]; maps locally sharpened using LocScale[5]. **e**, Angular distribution of particles that contributed to the final maps. The height of the cylinders is proportional to the number of particles assigned to each view (coloured blue to red for least and most populated views, respectively).

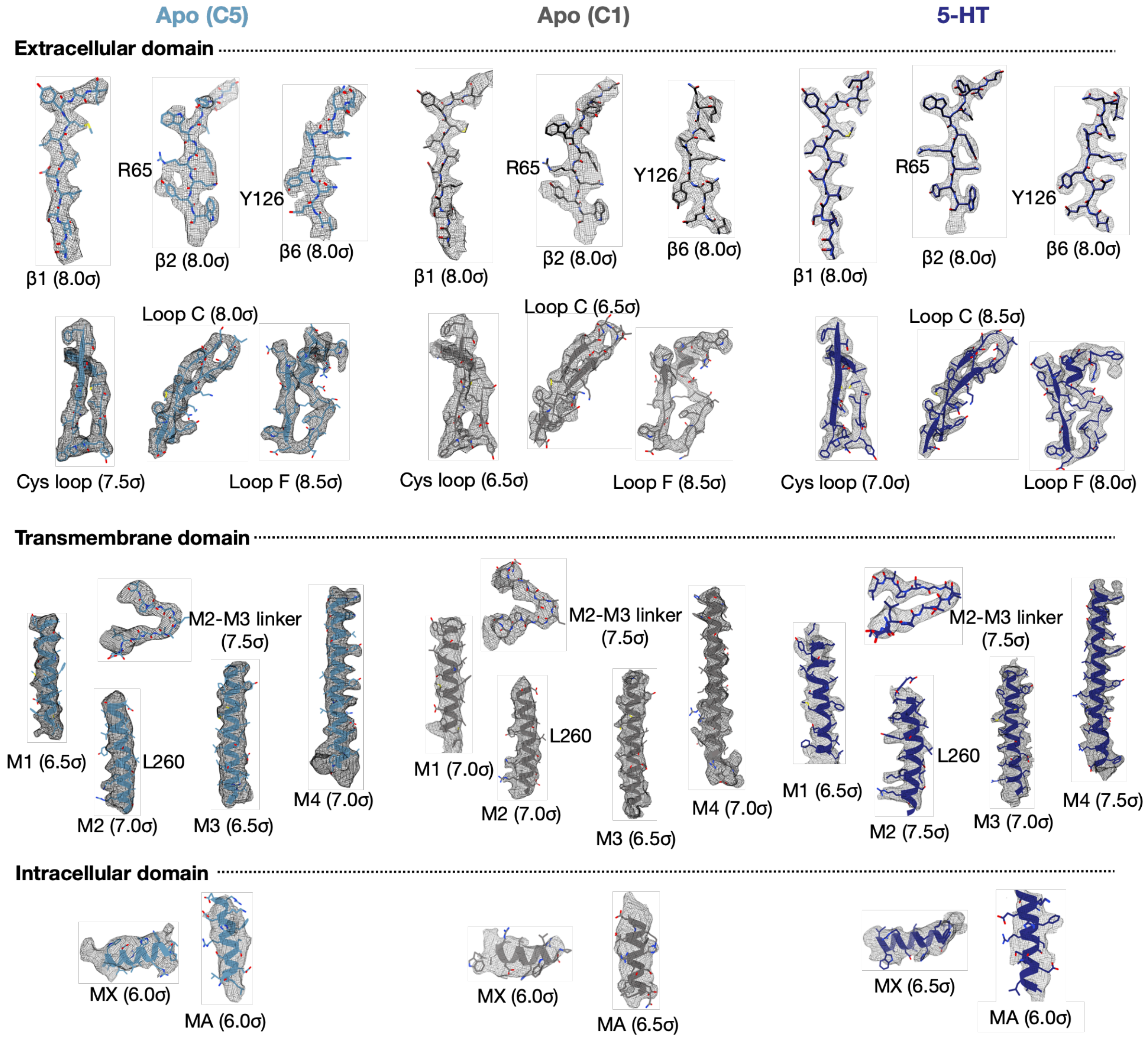

Supplementary Fig. 4. Map quality. Representative regions of the built apo-5HT$_3$R-Salipro (C5, light blue; C1, grey) and serotonin-5HT$_3$R-Salipro (dark blue) models and corresponding EM map densities locally sharpened using LocScale[5] (mesh) at the listed contour levels.

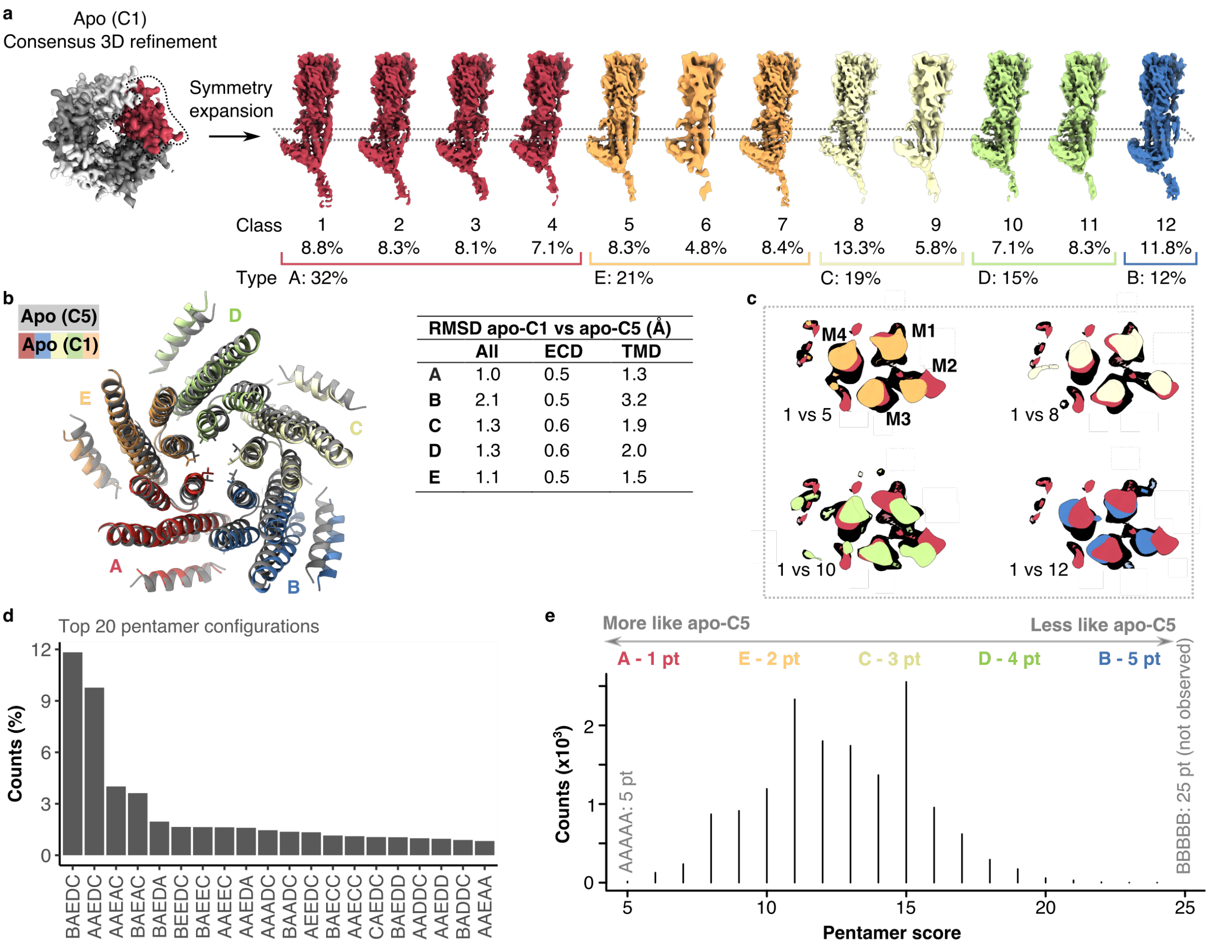

RMSD apo-C1 vs apo-C5 (Å)			
	All	ECD	TMD
A	1.0	0.5	1.3
B	2.1	0.5	3.2
C	1.3	0.6	1.9
D	1.3	0.6	2.0
E	1.1	0.5	1.5

Supplementary Fig. 5. Symmetry expansion and monomer-focused 3D classification of apo-C1 5HT$_3$R-Salipro data set. a, Symmetry expansion analysis of the apo-C1 5HT$_3$R-Salipro particle set: each pentamer was copied and rotated five times around its C5 (pseudo)symmetry axis, followed by signal subtraction to retain one copy of each monomer, and subsequent 3D classification without alignment into 12 classes. At the obtained resolution, all monomer classes could be divided into five different monomer groups (A-E) representing the five protomers resolved in the apo-C1 consensus map. The percentage of monomer particles corresponding to each group is also given. **b**, Top view of the TMD of the apo-C1 structure (coloured per chain) superimposed onto the apo-C5 structure (grey). The table gives the root mean square deviation (RMSD) between each apo-C1 subunit and the apo-C5 monomer for the whole chain (“All”), or the ECD or TMD domain only. **c**, Cross section of a superposition of the indicated monomer class onto class 1 at residue L260 (TMD) viewed from the ECD, highlighting the structural differences between the different types of classes. **d**, The subunit composition of the corresponding pentamers in the apo-C1 data set was investigated. Of the 629 possible different combinations, we observed 626 (not observed: BBBBB, BBBBC, DBBDB). The bar diagram shows the top 20 most populated pentamer configurations. The most prevalent pentamer configuration corresponded to the resolved consensus structure (“EDCBA”, 12% of pentamers). The top five configurations account for 31% of particles in total. Other conformations accounted for <1.7% each. **e**, The different apo-C1 monomer types were ranked from most to least deviating from the apo-C5 monomer as A < E < C < D < B based on backbone RMSD (see panel **b**). Each pentamer was scored based on its deviation from the apo-C5 structure accordingly: for every “A”, “E”, “C”, “D”, or “B”-type monomer present, 1 to 5 points were added, resulting in a score between 5 (5x“A”) and 25 (5x“B”, which was not resolved) for pentamers deviating least and most from the apo-C5 structure, respectively. A histogram of the scores is shown, illustrating that the distribution of these scores was skewed towards lower values (as also expected from the particle distribution between the classes).

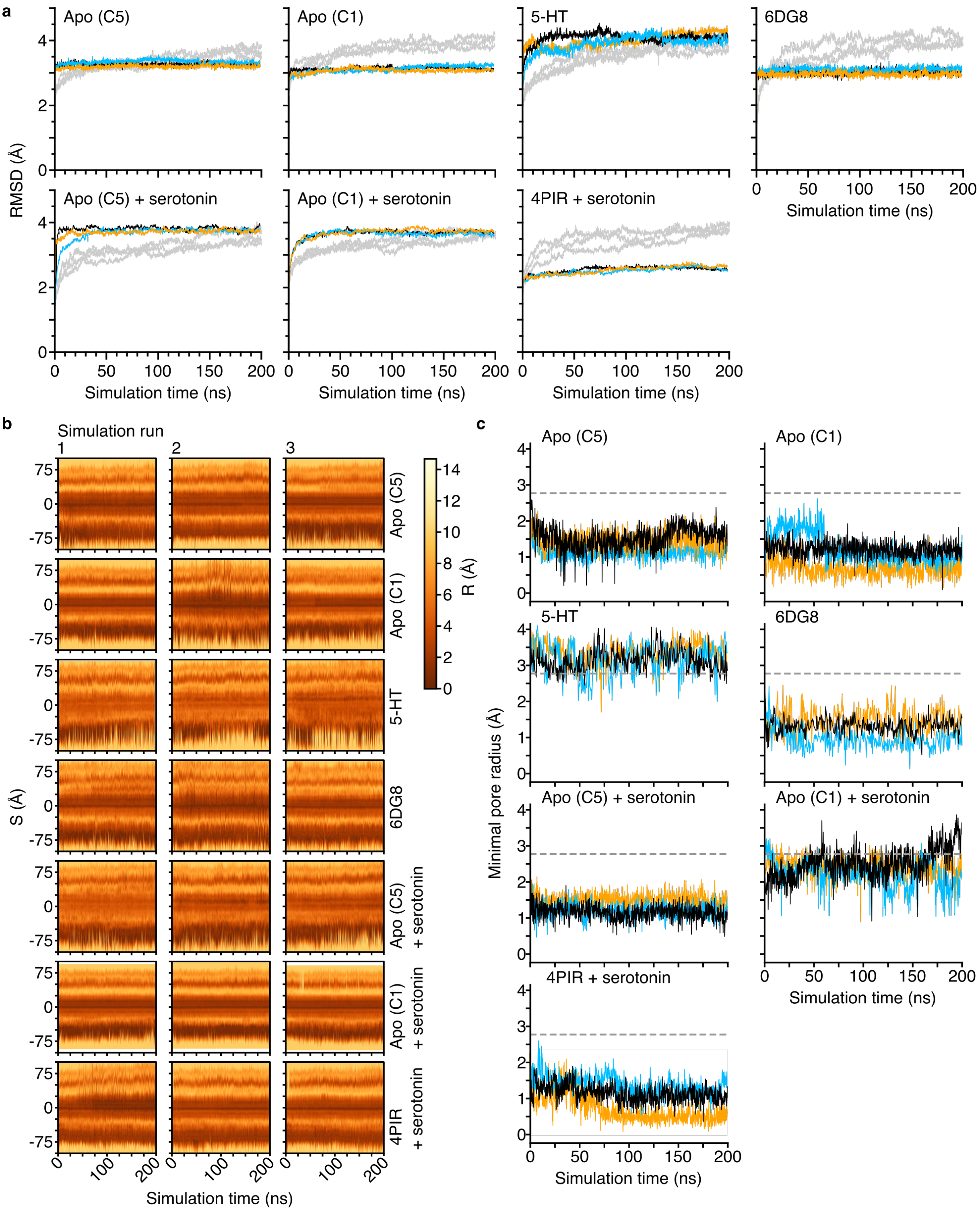

Supplementary Fig. 6. RMSD and pore radius during MD simulations. a, Backbone RMSD of the indicated models during the MD simulations. Solid lines in blue, orange, and black indicate results of three simulation runs, excluding the MX helices from the RMSD calculations; the MX helices highly flexible during the simulations due to the lack of an intrinsically disordered region (that is not included in the model) linking it to the intracellular domain (as also observed by Guros et al.[6]). Grey lines show backbone RMSD including the MX helices. **b**, Pore profile of the indicated models during the MD simulations, shown relative to the distance along the pore axis from the centre of mass of each model (S, with the ECD oriented towards positive values of S). **c**, Minimal pore diameter (for 40 Å TMD region) during MD simulations of the indicated models. The dashed grey line represents the radius of a hydrated sodium ion (2.76 Å).

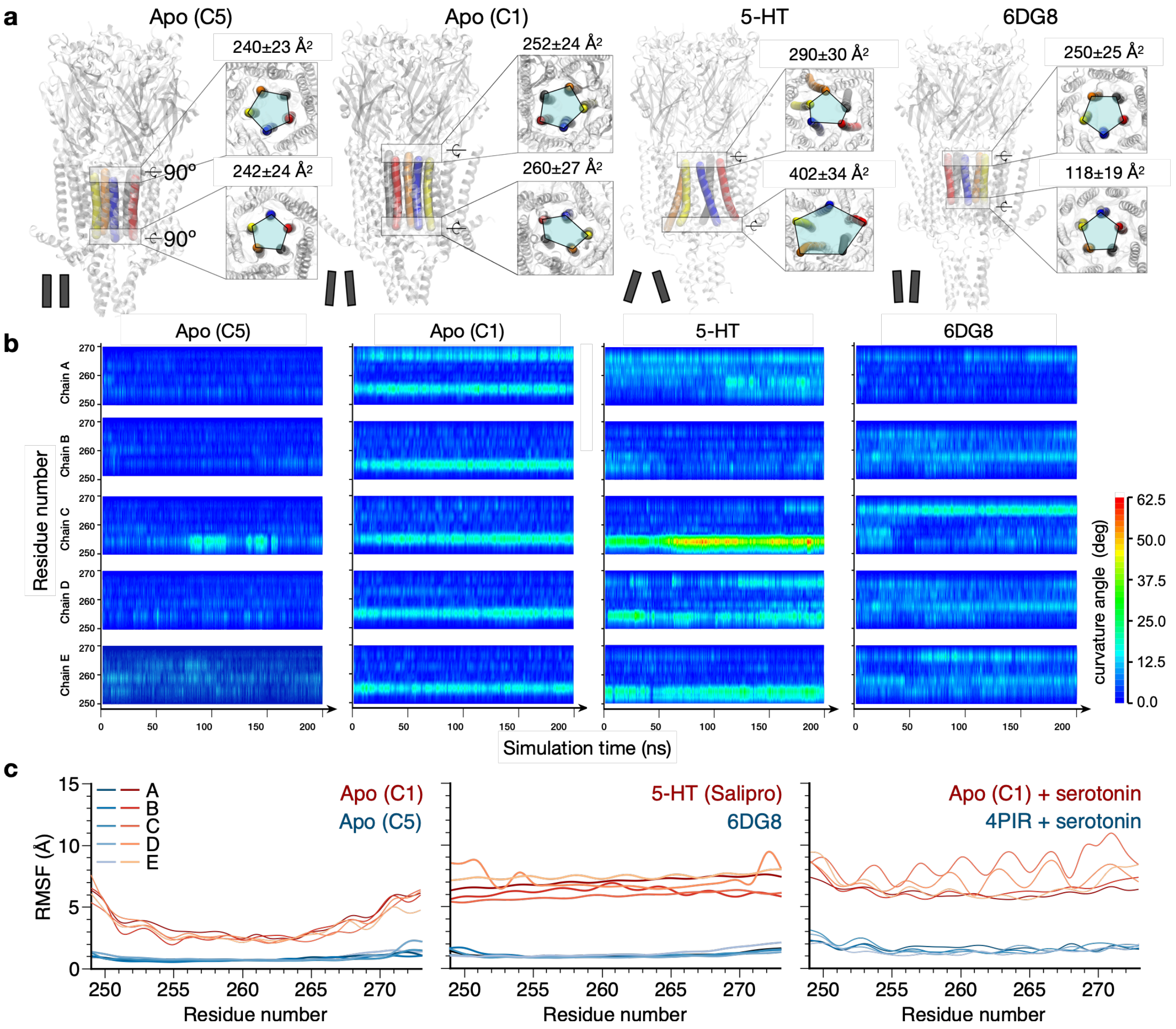

Supplementary Fig. 7. M2 conformation and pore wetting during MD simulations. a, Cross-sectional area at I268 (upper panels) and V252 (lower panels) showing different conformations of M2 for the apo-5HT$_3$R-Salipro (left, C5 and C1), serotonin-5HT$_3$R-Salipro (middle) and 6DG8 (right) models. **b**, Local curvature angle along M2 during the MD trajectories. **c**, Root-mean-square fluctuation (RMSF) of M2 for each chain (A-E) during MD simulations of the two apo-5HT$_3$R-Salipro models (left panel, C1 in red, C5 in blue), of serotonin-5HT$_3$R-Salipro and 6DG8 (middle panel, red and blue, respectively), and of the apo-C1 5HT$_3$R-Salipro and apo 4PIR models with serotonin molecules docked into each of the five ligand binding pockets (right panel, red and blue, respectively).

Supplementary Table 1. Lipid headgroup composition of 5HT$_3$R-Salipro. Lipid species (grouped by their headgroup moiety) extracted from 5HT$_3$R-loaded (5HT$_3$R-Salipro) and empty saposin-lipid discs as determined by lipidomics analysis. The lipid content is normalised to the molar amount of protein present (top) and to the molar amount of protein present as well as the total lipid content (bottom). Enrichment gives the factor by which the molar ratio of each lipid species differs in the 5HT$_3$R-Salipro sample relative to empty saposin-lipid discs; none of the lipid species shows strong positive enrichment in the receptor-containing discs compared to the empty discs. Results represent the mean (± standard deviation) of three and four biological replicates for 5HT$_3$R-Salipro and empty saposin-lipid discs, respectively. Source data are provided in the Source Data file. Abbreviations: PC, phosphatidylcholine; PE, phosphatidylethanolamine; PS, phosphatidylserine; PI, phosphatidylinositol; Chol, cholesterol; LPC, lyso-PC; SM, sphingomyelin; Cer, ceramide.

	**Lipid/protein (mol/mol)**	
	**5HT$_3$R-Salipro**	**Empty saposin discs**
**PC**	14±6	0.7±0.2
**PE**	7±2	0.5±0.1
**PS**	7±2	0.4±0.1
**PI**	4±3	0.16±0.10
**Chol**	19±8	1.0±0.4
**LPC**	0.06±0.02	0.028±0.009
**SM**	1.2±0.3	0.06±0.02
**Cer**	0.06±0.03	0.0023±0.0007

	**Molar ratio (%)**		**Enrichment**
	**5HT$_3$R-Salipro**	**Empty saposin discs**	
**PC**	30±10	30±7	1.0±0.5
**PE**	13±5	18±4	0.8±0.3
**PS**	14±4	13±3	1.0±0.4
**PI**	8±6	6±3	1±1
**Chol**	40±20	30±10	1.0±0.6
**LPC**	0.12±0.03	1.0±0.3	0.12±0.05
**SM**	2.3±0.7	2.0±0.6	1.2±0.5
**Cer**	0.11±0.05	0.08±0.03	1.3±0.7

Supplementary Table 2. Asymmetry of 5HT$_3$R-Salipro models. Backbone RMSD between **(1)** the individual chains of the apo-C1 5HT$_3$R-Salipro model and between each chain of the apo-C1 model and the apo-C5 model calculated for the entire chain, the ECD only, and the TMD only; and between **(2)** the individual chains of the serotonin-5HT$_3$R-Salipro model for the entire chain, and the ECD only.

**(1) Asymmetry apo-C1 5HT$_3$R-Salipro model**						
**Whole chain**	**RMSD (Å)**					
	**A**	**B**	**C**	**D**	**E**	**C5**
**A**	-	2.3	1.4	1.6	1.4	1.0
**B**	2.3	-	2.9	2.9	1.7	2.1
**C**	1.4	2.9	-	2.1	2.2	1.3
**D**	1.6	2.9	2.1	-	1.7	1.3
**E**	1.4	1.7	2.2	1.7	-	1.1
**ECD only**	**RMSD (Å)**					
	**A**	**B**	**C**	**D**	**E**	**C5**
**A**	**-**	0.4	0.5	0.5	0.4	0.5
**B**	0.4	-	0.5	0.5	0.5	0.5
**C**	0.5	0.5	-	0.4	0.4	0.6
**D**	0.5	0.5	0.4	-	0.5	0.6
**E**	0.4	0.5	0.4	0.5	-	0.5
**TMD only**	**RMSD (Å)**					
	**A**	**B**	**C**	**D**	**E**	**C5**
**A**	**-**	3.5	2.0	2.4	2.1	1.3
**B**	3.5	-	4.5	4.6	2.4	3.2
**C**	2.0	4.5	-	3.2	3.4	1.9
**D**	2.4	4.6	3.2	-	2.6	2.0
**E**	2.1	2.4	3.4	2.6	-	1.5

**(2) serotonin-5HT$_3$R-Salipro model**					
**Whole chain**	**RMSD (Å)**				
	**A**	**B**	**C**	**D**	**E**
**A**	-	3.5	1.9	3.1	2.8
**B**	3.5	-	4.0	1.9	2.0
**C**	1.9	4.0	-	3.4	3.2
**D**	3.1	1.9	3.4	-	1.9
**E**	2.8	2.0	3.2	1.9	-
**ECD only**	**RMSD (Å)**				
	**A**	**B**	**C**	**D**	**E**
**A**	**-**	0.8	0.7	0.7	0.7
**B**	0.8	-	0.7	0.8	0.7
**C**	0.7	0.7	-	0.7	0.7
**D**	0.7	0.8	0.7	-	0.7
**E**	0.7	0.7	0.7	0.7	-

Supplementary Table 3. Comparison between the TMD of the apo-C1, apo-C5, and serotonin-bound $5HT_3R$-Salipro models. The table give the displacements measured at the Cα atoms of residues on each helix in the middle of the TMD of the apo-C5, apo-C1, and serotonin-bound $5HT_3R$-Salipro models (cross section as shown in Fig. 8b). Displacements are derived from a superposition of the models that minimises the summed displacement of the indicated residues.

**Apo-C1 vs Apo-C5**					
	**Distance (Å)**				**Rotation (º)**
**Chains/TM**	**M1**	**M2**	**M3**	**M4**	
(Residue)	(V237)	(L260)	(V295)	(L442)	
**A**	0.7	1.9	1.0	1.0	1.0
**B**	2.7	2.8	3.4	3.2	5.9
**C**	1.1	1.8	1.4	0.6	2.8
**D**	3.5	3.8	1.5	1.6	0.3
**E**	0.5	1.9	2.2	0.7	1.5
**Sum**	8.5	12.2	9.5	7.1	-
	(Total: 37.3)				

**Apo-C1 vs serotonin-bound**					
	**Distance (Å)**				**Rotation (º)**
**Chains/TM**	**M1**	**M2**	**M3**	**M4**	
(Residue)	(V237)	(L260)	(V295)	(L442)	
**A**	5.2	7.0	4.5	2.0	14.0
**B**	4.2	4.2	2.5	1.8	7.7
**C**	4.3	6.9	3.8	1.1	17.9
**D**	8.3	8.1	3.3	4.0	22.2
**E**	4.2	5.4	2.8	1.4	15.1
**Sum**	26.2	31.6	16.9	10.3	-
	(Total: 85.0)				

**Apo-C5 vs serotonin-bound**					
	**Distance (Å)**				**Rotation (º)**
**Chains/TM**	**M1**	**M2**	**M3**	**M4**	
(Residue)	(V237)	(L260)	(V295)	(L442)	
**A**	4.7	7.7	4.5	1.3	19.7
**B**	5.0	5.1	3.9	4.2	12.3
**C**	5.1	8.3	5.1	1.6	21.1
**D**	4.8	4.7	2.7	2.8	10.5
**E**	4.2	4.7	3.1	2.1	10.6
**Sum**	23.8	30.8	19.3	12.0	-
	(Total: 85.9)				

Supplementary Table 4. TMD inter-subunit cavity. Volume of the TMD inter-subunit cavities for the apo-C5, apo-C1 and serotonin-bound (5-HT) forms as calculated by CASTp[7] analysis using a 1.4 Å probe sphere. For the asymmetric conformations, the size of each of the five pockets between the indicated chains is given.

Conformation	Subunits	Volume (Å^3)
**Apo-C5**	-	177
**Apo-C1**	A/B	428
	B/C	67
	C/D	352
	D/E	117
	E/A	40
**5-HT**	A/B	1001
	B/C	347
	C/D	1198
	D/E	702
	E/A	310

Supplementary Table 5. Details of data collection and model validation.

	**5-HT**	**Apo (C5)**	**Apo (C1)**
Data collection and processing			
Microscope	Titan Krios	Titan Krios	
Magnification	96,000	96,000	
Voltage (kV)	300	300	
Detector	Falcon III	Falcon III	
Exposure (e-/Å^2)	30	30	
Frames/movie	69	69	
Defocus range (μ)	-1.6 to -2.6	-1.6 to -2.6	
Pixel size (Å)	0.832	0.832	
Symmetry imposed	C1	C5	C1
Number of images	2,261*	5,028*	
Particles extracted (total)	642k	3.2M	
Particles refined (final)	251,610	238,701	152,877
Resolution unmasked (Å)	3.13	3.50	3.46
Resolution masked (Å)	2.77	3.17	3.10
FSC threshold	0.143	0.143	0.143
Model vs Map cross validation (Å)			
Model vs half map (work)	3.3	4.1	3.9
Model vs whole map	3.2	4.0	3.7
FSC threshold	0.5	0.5	0.5
Validation			
Ramachandran Plot			
Favored (%)	95.73	98.78	98.06
Allowed (%)	4.27	1.22	1.94
Disallowed (%)	0	0	0
Molprobity score	1.41 (100th percentile)	0.99 (100th percentile)	1.16 (100th percentile)
Molprobity clashscore	3.27 (100th percentile)	2.19 (100th percentile)	3.69 (100th percentile)
Poor rotamers (%)	0	0	0

(*Two data sets were collected on different dates for apo-5HT$_3$R-Salipro using the same grid with the same settings, and subsequently merged.)

Supplementary references

1. Smart, O. S., Neduvelil, J. G., Wang, X., Wallace, B. A. & Sansomt, M. S. P. HOLE: A program for the analysis of the pore dimensions of ion channel structural models. *J. Mol. Graph.* **7855,** 354–360 (1996).
2. Scheres, S. H. W. RELION: Implementation of a Bayesian approach to cryo-EM structure determination. *J. Struct. Biol.* **180,** 519–530 (2012).
3. Zivanov, J. *et al.* New tools for automated high-resolution cryo-EM structure determination in RELION-3. *Elife* **7,** e42166 (2018).
4. Tan, Y. Z. *et al.* Addressing preferred specimen orientation in single-particle cryo-EM through tilting. *Nat. Methods* **14,** 793–796 (2017).
5. Jakobi, A. J., Wilmanns, M. & Sachse, C. Model-based local density sharpening of cryo-EM maps. *Elife* **6,** e27131 (2017).
6. Guros, N. B., Balijepalli, A. & Klauda, J. B. Microsecond-timescale simulations suggest 5-HT–mediated preactivation of the 5-HT_{3A} serotonin receptor. *Proc. Natl. Acad. Sci. U. S. A.* **117,** 405–414 (2020).
7. Tian, W., Chen, C., Lei, X., Zhao, J. & Liang, J. CASTp 3.0: computed atlas of surface topography of proteins. *Nucleic Acids Res.* **46,** W363–W367 (2018).
