# Supplementary material for: Asymmetric opening of the homopentameric 5-HT3A serotonin receptor in lipid bilayers
Source: Nat Commun. 2021 Feb 16;12:1074. doi: 10.1038/s41467-021-21016-7 (PMC7887223; doi:10.1038/s41467-021-21016-7)
Supplement: Supplementary file 3 — Description of Additional Supplementary Files [file 41467_2021_21016_MOESM3_ESM.pdf]

Description of Additional Supplementary Files

Title: Supplementary Movie 1.

Description: Morph from the apo to serotonin-bound conformation for 5HT3R-Salipro and 5HT3R-detergent. The 5HT3R-Salipro models are coloured light blue, with chain C coloured dark blue. The 5HT3Rdetergent models are coloured yellow with chain C coloured orange. The symmetric apo conformation was used to prepare the morph of the 5HT3RSalipro model, and the 6BE1 and 6DG8 structures for the morph of the 5HT3R-detergent model.

Title: Supplementary Movie 2.

Description: MD simulation of a Na+ ion permeation event. The serotonin-5HT3R-Salipro model is shown in grey with pore-residues labelled and shown in blue. Red spheres represent water molecules. A sodium ion (yellow sphere) is observed to travel through the pore.
