# Supplementary material for: Asymmetric opening of the homopentameric 5-HT3A serotonin receptor in lipid bilayers
Source: Nat Commun. 2021 Feb 16;12:1074. doi: 10.1038/s41467-021-21016-7 (PMC7887223; doi:10.1038/s41467-021-21016-7)
Supplement: Supplementary file 6 — Reporting Summary [file 41467_2021_21016_MOESM6_ESM.pdf]

nature research

Corresponding author(s): M. Kudryashev, S. Yuan

Last updated by author(s): Dec 16, 2020

Reporting Summary

Nature Research wishes to improve the reproducibility of the work that we publish. This form provides structure for consistency and transparency in reporting. For further information on Nature Research policies, see our Editorial Policies and the Editorial Policy Checklist.

Statistics

For all statistical analyses, confirm that the following items are present in the figure legend, table legend, main text, or Methods section.

n/a	Confirmed	
☐	☒	The exact sample size (n) for each experimental group/condition, given as a discrete number and unit of measurement
☐	☒	A statement on whether measurements were taken from distinct samples or whether the same sample was measured repeatedly
☒	☐	The statistical test(s) used AND whether they are one- or two-sided<br>*Only common tests should be described solely by name; describe more complex techniques in the Methods section.*
☒	☐	A description of all covariates tested
☒	☐	A description of any assumptions or corrections, such as tests of normality and adjustment for multiple comparisons
☐	☒	A full description of the statistical parameters including central tendency (e.g. means) or other basic estimates (e.g. regression coefficient) AND variation (e.g. standard deviation) or associated estimates of uncertainty (e.g. confidence intervals)
☒	☐	For null hypothesis testing, the test statistic (e.g. F, t, r) with confidence intervals, effect sizes, degrees of freedom and P value noted<br>*Give P values as exact values whenever suitable.*
☒	☐	For Bayesian analysis, information on the choice of priors and Markov chain Monte Carlo settings
☒	☐	For hierarchical and complex designs, identification of the appropriate level for tests and full reporting of outcomes
☒	☐	Estimates of effect sizes (e.g. Cohen's d, Pearson's r), indicating how they were calculated

Our web collection on statistics for biologists contains articles on many of the points above.

Software and code

Policy information about availability of computer code

Data collection	A Titan Krios microscope (Thermo Fisher Scientific) operated at 300 kV and equipped with Falcon III (Thermo Fisher Scientific) using EPU software (Thermo Fisher Scientific) for automated data collection. Data collection quality was monitored using Warp (version 1.0.5).
Data analysis	Warp 1.0.5; MotionCor2; CTFFIND4.1; Gctf 1.06; RELION 3.0; cryoSPARC 2.8.0; UCSF pyem v0.4; Coot 0.9-pre; Phenix-1.12-2829; Molprobity 4.5; PSIPRED 4.0; LocScale; 3DFSC (processing webserver); HOLE v2.2.005; CASTp 3.0; UCSF Chimera 1.12; VMD v1.9.3; PyMOL v1.8.5.1 (beta); Schrödinger software suit (PROPKA tool, Epik tool); OPLS_2005 force field; CHARMM-GUI; CHARMM36m forcefield; CHARMM CgenFF force field; ORCA; M-SHAKE; GROMACS-2018.3; OPM web server (https://opm.phar.umich.edu/ppm_server); CHAP v0.9.1; PR. ThermChontrol 2.1.6; MO.Affinity 2.2.7; OriginPro 2015; Lipid Data Analyzer; Inkscape 0.91

For manuscripts utilizing custom algorithms or software that are central to the research but not yet described in published literature, software must be made available to editors and reviewers. We strongly encourage code deposition in a community repository (e.g. GitHub). See the Nature Research guidelines for submitting code & software for further information.

Data

Policy information about availability of data

All manuscripts must include a data availability statement. This statement should provide the following information, where applicable:

- Accession codes, unique identifiers, or web links for publicly available datasets
- A list of figures that have associated raw data
- A description of any restrictions on data availability

Atomic coordinates of the murine 5HT3R reconstituted into Salipro have been deposited at the Protein Data Bank (PDB) under accession codes 6Y5A (serotonin-bound), 6Y59 (apo-C5), and 6Y5B (apo-C1). The cryo-EM maps have been deposited at the Electron Microscopy Data Bank under accession codes EMD-10692 (serotonin-bound), EMD-10691 (apo-C5), and EMD-10693 (apo-C1). The original movies and final particle datasets have been deposited at the Electron Microscopy

Public Image Archive (EMPIAR, accession code EMPIAR-10381).

Field-specific reporting

Please select the one below that is the best fit for your research. If you are not sure, read the appropriate sections before making your selection.

- [x] Life sciences
- [ ] Behavioural & social sciences
- [ ] Ecological, evolutionary & environmental sciences

For a reference copy of the document with all sections, see nature.com/documents/nr-reporting-summary-flat.pdf

Life sciences study design

All studies must disclose on these points even when the disclosure is negative.

Sample size	No statistical methods were used to predetermine sample size. Lipidomics experiments were performed in three and four biological replicates for 5HT3R-Salipro and empty saposin-lipid discs, respectively; nanoDSF experiments were performed in two and four biological replicates for the detergent-solubilised and Salipro sample, respectively; MST experiments were performed in technical duplicates. Lipidomics experiments were performed in biological triplicates (n=3). Biochemical experiments were performed in technical duplicates (n=2).
Data exclusions	All cryo-EM data were included in initial analysis. A small number of movies was excluded from subsequent processing steps based on poor signal quality, excessive defocus, or particle movement, as these would be unlikely to improve the final EM maps.<br>A fourth biological replicate of the lipidomics experiment was excluded as the amount of sample used was 2-fold lower than in the other three biological repeats and the signal was not reliable.
Replication	No replication experiments beyond the reported biological/technical repeats were performed.
Randomization	Samples were not allocated into different groups (the same sample was used for structure determination with and without ligand added) and therefore randomisation is not applicable to our experiments
Blinding	Blinding was not applicable as we used predetermined samples and conditions in our experiments.

Reporting for specific materials, systems and methods

We require information from authors about some types of materials, experimental systems and methods used in many studies. Here, indicate whether each material, system or method listed is relevant to your study. If you are not sure if a list item applies to your research, read the appropriate section before selecting a response.

Materials & experimental systems

n/a	Involved in the study	
x		Antibodies
	x	Eukaryotic cell lines
x		Palaeontology and archaeology
x		Animals and other organisms
x		Human research participants
x		Clinical data
x		Dual use research of concern

Methods

n/a	Involved in the study	
x		ChIP-seq
x		Flow cytometry
x		MRI-based neuroimaging

Eukaryotic cell lines

Policy information about cell lines

Cell line source(s)	A stable T-Rex-293 cell line used for the expression of 5HT3R was provided by H. Vogel. The original T-Rex-293 cell line was obtained from Invitrogen.
Authentication	No authentication was performed.
Mycoplasma contamination	The cell line tested negative for mycoplasma contamination.
Commonly misidentified lines (See ICLAC register)	No commonly misidentified cell lines were used.
